# Real-Life Experience of Regorafenib in Patients With Advanced Hepatocellular Carcinoma

**DOI:** 10.3389/fphar.2022.917384

**Published:** 2022-06-06

**Authors:** Jing-Yu Hou, Ya-ting Xiao, Jing-Bo Huang, Xin-Hua Jiang, Kai Jiang, Xun Li, Li Xu, Min-Shan Chen

**Affiliations:** ^1^ Department of Liver Surgery, Sun Yat-sen University Cancer Center, Sun Yat-sen University, Guangzhou, China; ^2^ Collaborative Innovation Center for Cancer Medicine, State Key Laboratory of Oncology in South China, Guangzhou, China; ^3^ School of Molecular Medicine, Hangzhou Institute for Advanced Study, UCAS, Hangzhou, China; ^4^ Department of Hepatobiliary Surgery, The First Affiliated Hospital of Hunan Normal University (Hunan Provincial People's Hospital), Changsha, China; ^5^ Department of Radiology, Sun Yat-sen University Cancer Center, State Key Laboratory of Oncology in South China, Collaborative Innovation Center for Cancer Medicine, Guangzhou, China; ^6^ Department of Orthopaedics, The Second Xiangya Hospital, Central South University, Changsha, China; ^7^ School of Chemical Engineering, University of Chinese Academy of Sciences, Beijing, China

**Keywords:** hepatocellular carcinoma, prognosis, regorafenib, sorafenib, retrospective study

## Abstract

**Background:** The RESORCE trial reported that regorafenib was effective as the second-line treatment for patients with hepatocellular carcinoma (HCC) after progression on sorafenib. Real-world data are needed to assess clinical outcomes and adverse events in the setting of daily practice.

**Objective:** We aimed to evaluate the efficacy and safety of regorafenib after disease progression with sorafenib in Chinese patients with advanced HCC.

**Patients and Methods:** A total of 41 patients with advanced HCC who did not respond to sorafenib and followed a regorafenib regimen were enrolled in this retrospective study. Overall survival (OS), progression-free survival (PFS), radiological responses, and adverse events (AEs) were evaluated. Survival curves were compared by using the log-rank test and constructed with the Kaplan–Meier method.

**Results:** The median PFS with regorafenib was 6.6 months (range: 5.0–8.2 months), and the median OS with regorafenib was not reached. The 1-year OS rate of regorafenib was 66.4%. The median OS of sequential sorafenib to regorafenib treatment was 35.3 months [95% confidence interval (CI), 24.3–46.3], and the 2-year OS rate of sequential sorafenib to regorafenib treatment was 74.4%. The most common AEs of regorafenib treatment were elevated aspartate aminotransferase [17/41 patients (41.5%)], elevated alanine aminotransferase [16/41 patients (39%)] and hand-foot syndrome [14/41 patients (34.1%)].

**Conclusion:** Regorafenib appears to be safe and clinically effective in patients with advanced HCC who progressed on first-line sorafenib.

## Introduction

In 2018, liver cancer became the sixth most common cancer and the fourth leading cause of cancer-related deaths worldwide, with China ranking first worldwide in terms of the incidence of liver cancer (Bray et al., 2018). Due to the early characteristic symptoms and signs are not obvious, most patients with hepatocellular carcinoma (HCC) are often diagnosed at an advanced stage (Truty and Vauthey, 2010). Currently, for patient with advanced HCC have the following variable treatment modalities: transcatheter arterial chemoembolization (TACE), chemotherapy, and targeted drug therapy (Liccioni et al., 2014). About 80% of patients with advanced HCC who have unresectable tumors, and many are not diagnosed until their tumors have grown to a large (>5.0 cm) or very large size (>10 cm). Molecular targeted drugs such as sorafenib have been shown to significantly extend overall survival (OS) and time to progression (TTP) in patient with advanced HCC (Truty & Vauthey, 2010; (Yeo et al., 2005; (Palmer, 2008). Although sorafenib has been the main treatment for advanced HCC in the past decade, the emergence of drug resistance is still inevitable (Huang et al., 2020). For HCC patients whose disease progresses after sorafenib treatment, second-line oral regorafenib can significantly improve overall survival (Bruix et al., 2017; (Duffy and Greten, 2017; (Bruix et al., 2013).

Regorafenib is an oral multikinase inhibitor that blocks the activation of multiple angiogenesis kinases and oncogenic kinases, including vascular endothelial growth factor receptors (VEGFR 1, VEGFR2, and VEGFR3), platelet-derived growth factor receptor β, and fibroblast growth factor receptor 1, and mutated oncogenic kinases RAS, MAPK, and KIT (Wilhelm et al., 2011; (Subramonian et al., 2020). Compared with sorafenib, regorafenib targets a wider range of kinases, and has a stronger pharmacological effect (Strumberg and Schultheis, 2012). However, the RESORCE trial did not report the baseline clinical data of patients when sorafenib treatment was initiated. We need real-world data to learn more about the differences between daily practice and clinical trials in patients. Regorafenib was approved for HCC in China in 2017. Therefore, in this study we aimed to evaluate the safety and efficacy of regorafenib after disease progression with sorafenib in Chinese patients with advanced HCC, with the aim of complementing phase III findings.

## Methods

### Study Population Selection and Regorafenib Treatment

The study was a single-center, single-arm study. Patients were enrolled who met the following criteria: 1) patients were 18–80 years of age and had confirmed advanced HCC; 2) none of the patients had a history of other malignant tumors before the discovery of HCC; 3) complete clinical, imaging and follow-up data of the patients are available. The exclusion criteria were as follows: 1) patients who were given regorafenib for less than one medication cycle; 2) patients with any of the following conditions within 12 months prior to taking the drug: myocardial infarction, severe/unstable angina, coronary artery bypass grafting, congestive heart failure, cerebrovascular accident (including transient ischemic attack), pulmonary embolism; 3) patients with other severe, acute, chronic physical illness that may increase the risk associated with participating in study treatment, or may not be considered appropriate for inclusion by the investigator; and 4) patients with an expected survival time of less than 3 months. The complete eligibility criteria are shown in the supplementary data.

After screening, we retrospectively collected clinical data of patients with advanced HCC who received sequential sorafenib-regorafenib treatment at our center before February 2019. Patients with radiological progression during sorafenib therapy are strongly recommended for treatment with regorafenib. A total of 41 patients in our center were enrolled in this study, and each cycle included 4 weeks. They took 160 mg regorafenib per day for the first 3 weeks, and stopped all the treatment in the last week of the cycle (Bruix et al., 2017). Dose adjustment of regorafenib was allowed depending on patient tolerance.

### Clinical Parameters and Evaluation

We collected clinical parameters such as etiology, age, sex, Child-Pugh class, alpha-fetoprotein (AFP), metastasis of primary HCC, macrovascular invasion, treatment prior to or combined with regorafenib and sorafenib initiation, initial and final sorafenib and regorafenib dosses, adverse events (AEs) after treatment with sorafenib and regorafenib, date of radiological progression, and date of death or last follow-up. Efficacy was evaluated every 2 months by computed tomography (CT) or magnetic resonance imaging (MRI) scans and blood indicator (AFP level) assessment. All patients received contrast-enhanced CT or MRI examinations, unless the administration of the contrast material was contraindicated. PET-CT was generally performed when systemic progression needed to be evaluated. All images were assessed by one of the authors (J.X.H) who had 10 years of experience. Tumor imaging response and disease progression were evaluated according to the Response Evaluation Criteria in Solid Tumors (RECIST) version 1.1 (Eisenhauer et al., 2009). Reported AEs were assessed in terms of type, causality, and severity as graded by Common Terminology Criteria for Adverse Events (CTCAE) Version 5.0.

### Statistical Analysis

All study patients who met the eligibility criteria at baseline were included in the analyses. The primary endpoint for the study was OS and the secondary endpoint was progression-free survival (PFS). OS for regorafenib was defined as the time from the treatment of regorafenib to death from any cause. For sequential treatment with sorafenib-regorafenib, OS was defined as the time from the treatment of sorafenib to death from any cause. PFS was defined as the time from the initiation of regorafenib to the date of radiological assessment progression, or death. TTP was defined as the time from the initiation of sorafenib or regorafenib to the date of radiological assessment progression. OS, PFS, and TTP were estimated by using the Kaplan-Meier method with 95% confidence intervals (CIs). OS, PFS, and TTP were compared between different subgroups by means of the log-rank test. Analysis was performed using SPSS statistical software (version 24; SPSS-IBM, Chicago, IL, United States). *p* < 0.05 was considered statistically significant.

## Results

### Patient Characteristics

A total of 41 patients with advanced HCC who did not respond to sorafenib and followed a regorafenib regimen were enrolled in our study. The median age was 42 (range: 25–75) years; Among them, 33 (80.5%) patients were male. Most patients had a history of local treatment prior to regorafenib included 25 (61.0%) patients undergo radiofrequency ablation, 40 (97.6%) patients received interventional therapy [include transarterial chemoembolization (TACE) and transcatheter arterial infusion (TAI)] and eight patients (19.5%) received radiation therapy (RT). There were 9 (20.9%) patients had macrovascular invasion and 23 (53.5%) patients had extrahepatic metastasis. Most patients (78.0%) received a full dosage of sorafenib (800 mg). After sorafenib treatment failed, 28 patients (68.0%) received 160 mg once daily and 13 (32.0%) patients received 120 mg once daily as the starting dose of regorafenib. Of the 41 patients, 40 received other therapies as follows: TACE (*n* = 34), radiofrequency ablation (RFA) (*n* = 25), radiation therapy (*n* = 8), and transcatheter arterial infusion (TAI) (*n* = 1) during sequential sorafenib-regorafenib treatment. The baseline characteristics of patient are summarized in Table 1.

**TABLE 1:** Baseline characteristics of patients with hepatocellular carcinoma treated with regorafenib after sorafenib (*n* = 41).

Characteristics	Patients
Age, years, median (range)	41 (31–80)
Sex, male, *n* (%)	33 (80.5)
Etiology, *n* (%)	
Hepatitis B virus	40 (97.6)
Hepatitis C virus	0 (0)
Alcohol	6 (14.6)
Unknown	1 (2.4)
BCLC stage, *n* (%)	
B	16 (39.0)
C	25 (61.0)
ECOG, 0/1/2, *n*	18/22/1
Child-Pugh class, *n* (%)	
A	25 (61.0)
B	16 (39.0)
Extrahepatic metastasis, *n* (%)	23 (53.5)
Macrovascular invasion, *n* (%)	9 (20.9)
AFP≥400 ng/ml, *n* (%)	20 (48.8)
Therapies prior regorafenib, *n* (%)	
Resection	36 (87.8)
Radiofrequency ablation	25 (61.0)
TACE	34 (82.9)
TAI	6 (14.6)
Radiation therapy	8 (19.5)
Sorafenib	41 (100)
Tumor number, *n* (%)	
≥3	9 (22.0)
<3	32 (78.0)
Tumor diameter, median (range),cm	3.3 (1.0–9.8)
TTP of sorafenib (month)	7.0
Tumor progression patterns of sorafenib, *n* (%)	
New intrahepatic lesion	11 (26.8)
Increase in intrahepatic tumor size	15 (36.6)
Increase in extrahepatic tumor size/new extrahepatic lesion	15 (36.6)

BCLC, barcelona clinic liver cancer; ECOG, eastern cooperative oncology group; TACE, transarterial chemoembolization; AFP, Alpha-fetoprotein; TAI, transcatheter arterial infusion; TTP, Time to progression.

### Efficacy of Regorafenib

**FIGURE 1:**
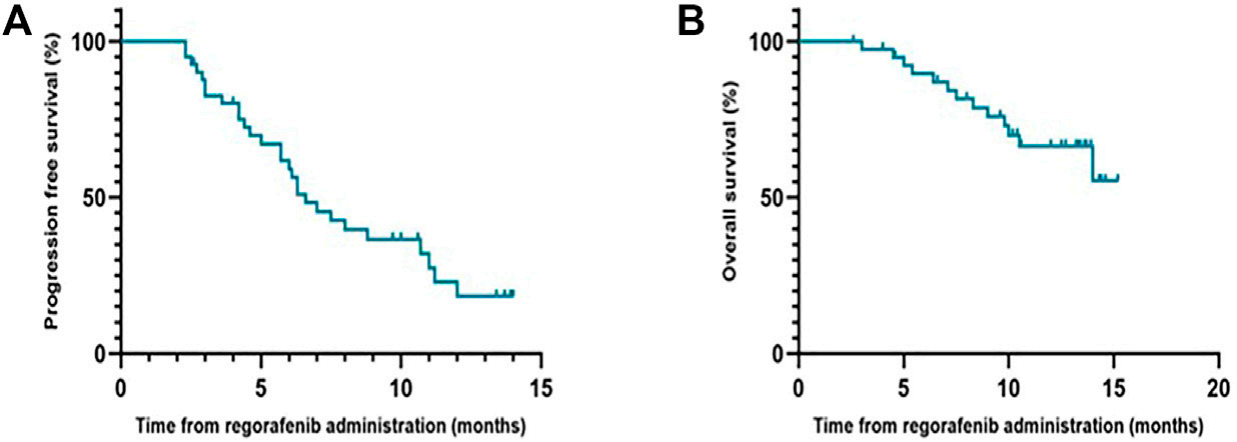
Progression-free survival **(A)** and Overall survival **(B)** of regorafenib in patients with advanced hepatocellular carcinoma in Chinese clinical settings.

**FIGURE 2:**
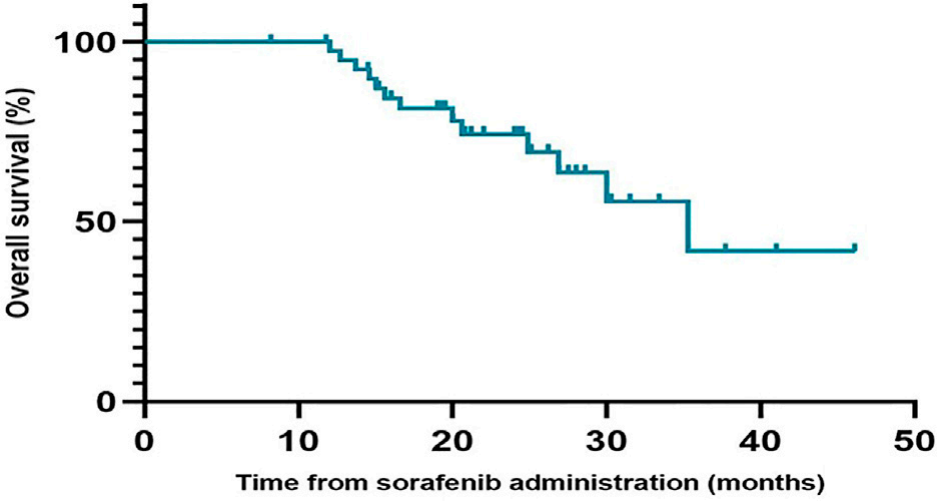
Overall survival of patients receiving sequential sorafenib-regorafenib treatment.

During the follow-up period, the median PFS of patients who received regorafenib after sorafenib was 6.6 months (95% CI, 5.0–8.2 months), and the median OS was not reached (Figure 1). The 1-year OS rate of patients who received regorafenib after sorafenib was 66.4% (95% CI, 50.72–82.08%). The median OS of patients receiving sequential sorafenib-regorafenib treatment was 35.3 months (95% CI, 24.3–46.3) (Figure 2), and the 2-year OS rate of patients receiving sequential sorafenib-regorafenib treatment was 74.4% (95% CI, 59.7–89.1%). The objective response and disease control rate during treatment with regorafenib were 9.8% (*n* = 4) and 80.5% (*n* = 33), respectively (Table 2). In this study, the response was indicated at stable disease in 29 patients (70.7%) and progressive disease in eight patients (19.5%). OS was associated with baseline alpha fetoprotein levels (<400 vs. ≥ 400 ng/ml; *p* = 0.049), but not with extrahepatic metastasis (*p* = 0.844), the starting dose of regorafenib (160 mg or <160 mg) (*p* = 0.615), or the last dose of sorafenib (800 mg or <800 mg) (*p* = 0.172). However, we found that the PFS had a relationship with the starting dose of regorafenib (160 mg or <160 mg) (10.7 vs. 5.7 months, *p* = 0.006), but not with the starting dose of sorafenib (800 mg or <800 mg) (8.0 vs. 5.7 months, *p* = 0.084), baseline alpha fetoprotein levels (<400 vs. ≥ 400 ng/ml; *p* = 0.108), or extrahepatic metastasis (*p* = 0.107).

**TABLE 2:** Efficacy of regorafenib treatment.

Variable	Total (*n* = 41)
Response by RECIST v 1.1	
Complete response	0
Progressive disease	8 (19.5%)
Stable disease	29 (70.7%)
Objective response rate	4 (9.8%)
Disease control rate	33 (80.5%)
Progression-free survival, median	6.6 months (95% CI, 5.0–8.2 months)
Overall survival, median	Not reached
One-year overall survival rate	66.4% (95% CI, 50.72–82.08%)

CI, confidence interval.

**FIGURE 3:**
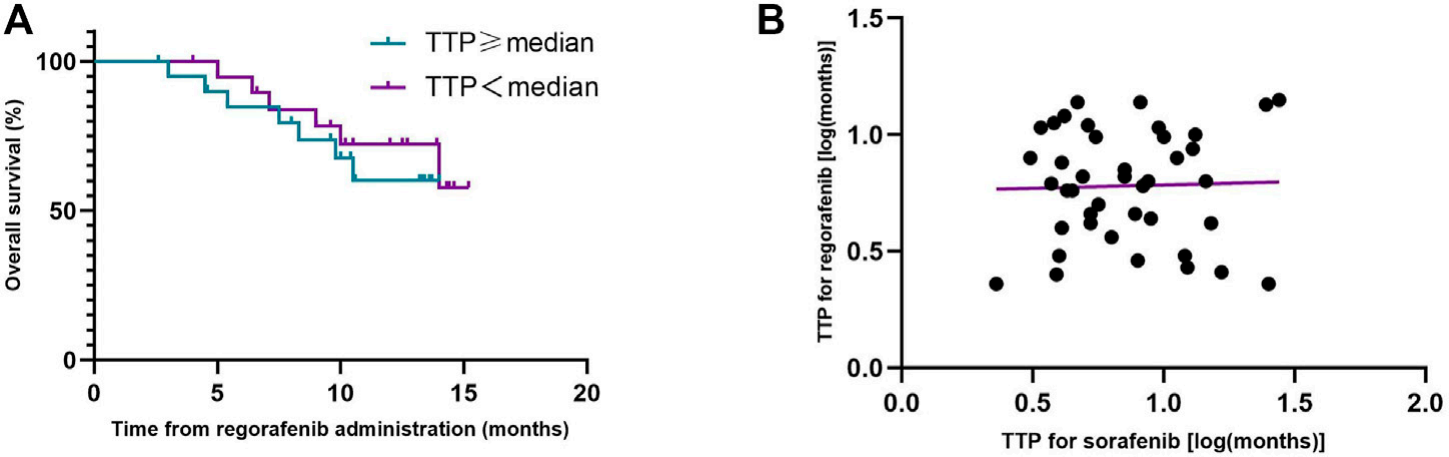
Kaplan-Meier analyses of overall survival during treatment with regorafenib according to the time to progression (TTP) on prior sorafenib treatment **(A)**, and correlation diagram of TTP between sorafenib and regorafenib **(B)**.

**FIGURE 4:**
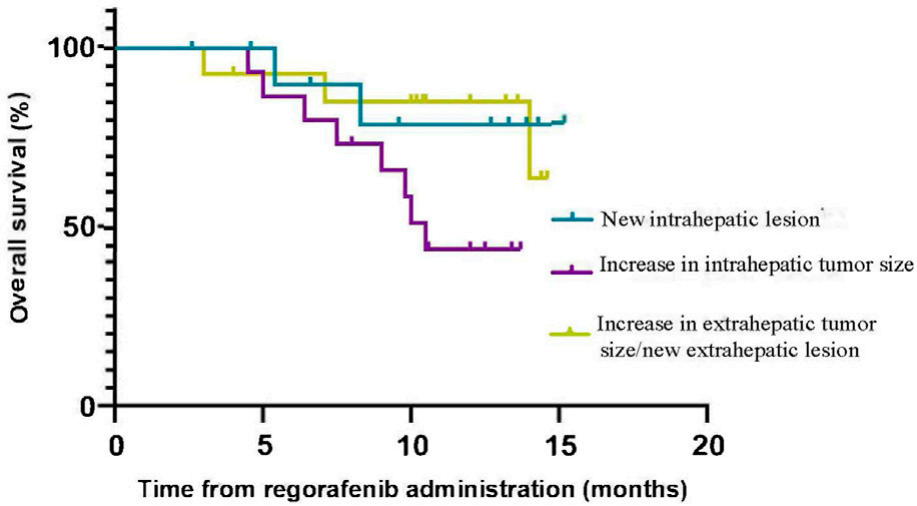
Overall survival of patients with three tumor progression patterns of sorafenib.

The median TTP (mTTP) with sorafenib was 7.0 months (95%CI, 4.2–9.8 months, suggesting that the mTTP with sorafenib is not relevant to the median OS of regorafenib (*p* = 0.552) (Figure 3A). We did not find any connection between these factors (R squared: 0.001) (Figure 3B). When we analyzed the tumor progression patterns of sorafenib, we found that the patients with an increase intrahepatic tumor size had the worst prognosis among those with disease progression (Figure 4).

### Safety and Tolerability of Regorafenib and Correlation of Adverse Events Between Sorafenib and Regorafenib

During the observation period, there were no treatment-related deaths from sorafenib and regorafenib. The most common cause of regorafenib dose modification was hand-foot skin reaction in four patients (9%), and regorafenib doses were reduced to 120 mg or 80 mg. However, two patients increased the regorafenib dose from 120 to 160 mg due to disease progression. During the follow-up period, 33 patients had at least one treatment-related AEs, and the most common AE during regorafenib treatment were elevated aspartate aminotransferase [17/41 patients (41.5%)], elevated alanine aminotransferase [16/41 patients (39%)] and hand-foot syndrome [14/41 patients (34.1%)] (Table 3).

**TABLE 3:** Adverse events (AEs) of regorafenib treatment (>10% of patients).

Adverse events	Any grades, *n* (%)
Treatment related AEs	33 (80.5)
Palmar-plantar erythrodyses-thesia	14 (34.1)
Diarrhea	12 (29.3)
Abdominal distension	5 (12.2)
Decreased appetite	6 (14.6)
Elevated aspartate aminotransferase	17 (41.5)
Elevated alanine aminotransferase	16 (39.0)
Hypertension	5 (12.2)

**FIGURE 5:**
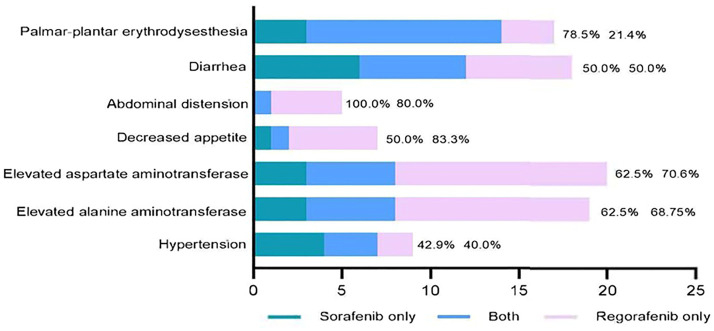
Correlation of common adverse events between sorafenib and regorafenib in patients with advanced hepatocellular carcinoma. Reproducibility rates of regorafenib related adverse events during sorafenib therapy (left side) and occurrence rates of regorafenib related adverse events which did not found during sorafenib therapy (right side) are indicated in this figure.

We also compared the adverse events during sorafenib and regorafenib treatment in 41 patients (Figure 5). All common sorafenib-related adverse events also emerged during regorafenib therapy. The most common adverse events that were observed during both sorafenib and regorafenib therapy were hand-foot syndrome and diarrhea. Conversely, 21.4% of the patients who did not have palmar-plantar erythrodyses-thesia during sorafenib therapy developed this adverse event during regorafenib therapy.

## Discussion

This study was a single center, retrospective analysis of Chinese patients who received regorafenib after progression disease of sorafenib. The results of this retrospective analysis not only demonstrate the efficacy and safety of sequential sorafenib-regorafenib therapy in Chinese patients with advanced HCC but also provide detailed clinical data that the RESORCE trial that did not provide. These results provide the first outcome data for sequential sorafenib-regorafenib treatment in patients with advanced HCC in China.

There was no linear relationship between the TTP of sorafenib and regorafenib. (R squared: 0.001). The result is similar to additional analyses from the phase III RESORCE trial (Finn et al., 2018). However, a study in Japan showed that after sorafenib, the group with TTP >4.6 months had a significantly longer TTP during regorafenib therapy than the group with TTP ≤4.6 months (Ogasawara et al., 2020). We compared the two groups of data and found that sorafenib had no significant effect on a group of Japanese patients, and disease progression occurred soon after the use of the drug. Even if they changed to regorafenib, the drug had no significant effect. However, this did not occur in Chinese patients, who responded differently to the two drugs.

In our cohort, the patients could adjust the dosage according to their tolerance, nine patients took sorafenib less than 800 mg every day, and 13 patients took regorafenib less than 160 mg every day. They chose dosage reduction because of AEs. The most frequent AEs during sorafenib treatment were hand-foot syndrome, diarrhea and elevated aspartate aminotransferase, but the most frequent AEs during regorafenib treatment were elevated aspartate aminotransferase, elevated alanine aminotransferase and hand-foot syndrome. Both drugs are oral multikinase inhibitors, so the patients had overlapping adverse-event profiles, and their tolerability to regorafenib was improved after treatment with sorafenib (Heo and Syed, 2018). The majority of these events were lower than grade 3 and can be alleviated by expectant treatment or by reducing the treatment dose. For example, the rate of hand-foot syndrome in our study was higher than that in the RESORCE trial, but we always advise the patients to use hand cream containing salicylic acid and take celecoxib or minocycline hydrochloride capsules to treat hand-foot syndrome (Rimassa et al., 2019; (Chen et al., 2020). Dose personalization of drugs and minimizing side effects could improve their tolerability to drugs (Rizzo et al., 2020).

In addition, we analyzed the tumor progression pattern and found that there were three tumor progression patterns of sorafenib: increase in intrahepatic tumor size, new intrahepatic lesion, and increase in extrahepatic tumor size/new extrahepatic lesion. The results suggested that patients with an increase in intrahepatic tumor size had the worst prognosis. This result is different from another study in which the prognosis of patients with radiologic tumor progression due to an increase in extrahepatic tumor size/new extrahepatic lesion (NEH) was the worst (Reig et al., 2013). When comparing the results of the previous imaging data with new imaging data, we also found that patients’ liver function deteriorated (elevated aspartate aminotransferase or alanine aminotransferase, increased total bilirubin, hypoproteinemia, ascites), when intrahepatic tumor size increased or new intrahepatic lesion occurred. But we are unable to treat patients with antitumor therapy because of their poor liver function (Terashima et al., 2018). In the end, antitumor therapy was not available for these patients, so their survival time was short. It is critical to preserve liver function so that patients are candidates for any antitumor therapy, as patients with poor performance status cannot obtain any survival benefit from HCC-directed therapy (Kirstein et al., 2020; (Piscaglia and Ogasawara, 2018; (Rich et al., 2017).

The median OS from initiation of sorafenib to regorafenib was 35.3 months, which was longer than that observed in the RESORCE trial. We compared the differences between the two groups, and the basic condition in our group was different from that in the RESORCE trial. For example, the proportion of patients with macrovascular invasion or BCLC stage C was lower than that in the RESORCE trial, and most patients in the RESORCE trial had extrahepatic disease. One study showed that among patients with advanced HCC after sorafenib, few patients with MVI or hypoalbuminemia at sorafenib initiation were able to undergo regorafenib treatment (Uchikawa et al., 2018). Hepatitis B is the main cause of HCC in China, and the majority of HCC patients have large HCC tumors (Truty and Vauthey, 2010; (Chen et al., 2019). The main cause of HCC in Europe and America is fatty liver and alcoholic liver disease, and the majority of HCC patients have small HCC tumors. This is also a basic difference between Chinese patients with HCC and HCC patients from other countries, so there are also differences in the treatment effect. Furthermore, our patients could receive many other treatments such as RFA, TACE, RT, and TAI, during sequential sorafenib-regorafenib treatment, and combining local therapies (RFA, TACE, RT) or systemic therapy (TAI) with drug treatment might have the potential to prolong OS. The variety of new treatments for patients with advanced HCC who do not respond to sorafenib means that multidisciplinary management could obtain an effect (Liu et al., 2018).

Furthermore, a series of exciting breakthroughs in HCC treatment will bring survival benefits to the majority of patients, and a revolution in advanced HCC treatment has been driven by combined therapy and immunotherapy in recent years (El-Khoueiry et al., 2017; (Kudo, 2018). The combination of regorafenib and immunotherapy drugs has also demonstrated synergistic antitumor effect (Wu et al., 2019; (Tsai et al., 2017; (Von Felden, 2020). Therefore, the combination of regorafenib and immunotherapy for HCC may be a good choice.

There are some limitations in our study need to be considered. Firstly, this study was retrospectively and carried out without randomization which may have resulted in selection bias and confounding. Second, as a single center study with small sample size, our results are limited in terms of generalization. The results of our study need further validation in the large-scale multi-center study and more randomized controlled trials.

## Conclusion

In summary, the results of this retrospective analysis verified the efficacy and safety of regorafenib in patients with advanced HCC in China. Regorafenib combined with other treatments may bring survival benefits to patients. However, this is a retrospective and single-arm study without a control group. Moreover, this retrospective study had small sample size. Additional large-scale studies are needed to explore and expound on the specific treatment plan for patients.

## Data Availability

The original contributions presented in the study are included in the article/supplementary material, further inquiries can be directed to the corresponding authors.

## References

[B1] Bray F. Ferlay J. Soerjomataram I. Siegel R. L. Torre L. A. Jemal A. (2018). Global Cancer Statistics 2018: GLOBOCAN Estimates of Incidence and Mortality Worldwide for 36 Cancers in 185 Countries. CA Cancer J. Clin. 68, 394–424. 10.3322/caac.21492 30207593

[B2] Bruix J. Qin S. Merle P. Granito A. Huang Y. H. Bodoky G. (2017). Regorafenib for Patients with Hepatocellular Carcinoma Who Progressed on Sorafenib Treatment (RESORCE): a Randomised, Double-Blind, Placebo-Controlled, Phase 3 Trial. Lancet 389, 56–66. 10.1016/s0140-6736(16)32453-9 27932229

[B3] Bruix J. Tak W.-Y. Gasbarrini A. Santoro A. Colombo M. Lim H.-Y. (2013). Regorafenib as Second-Line Therapy for Intermediate or Advanced Hepatocellular Carcinoma: Multicentre, Open-Label, Phase II Safety Study. Eur. J. Cancer 49, 3412–3419. 10.1016/j.ejca.2013.05.028 23809766

[B4] Chen J. C. Wang J. C. Pan Y. X. Yi M. J. Chen J. B. Wang X. H. (2020). Preventive Effect of Celecoxib in Sorafenib-Related Hand-Foot Syndrome in Hepatocellular Carcinoma Patients, a Single-Center, Open-Label, Randomized, Controlled Clinical Phase III Trial. Am. J. Cancer Res. 10, 1467–1476. 32509392 PMC7269790

[B5] Chen W. Xia C. Zheng R. Zhou M. Lin C. Zeng H. (2019). Disparities by Province, Age, and Sex in Site-specific Cancer Burden Attributable to 23 Potentially Modifiable Risk Factors in China: a Comparative Risk Assessment. Lancet Glob. Health 7, e257–e269. 10.1016/s2214-109x(18)30488-1 30683243

[B6] Duffy A. G. Greten T. F. (2017). Liver Cancer: Regorafenib as Second-Line Therapy in Hepatocellular Carcinoma. Nat Rev Gastroenterol HepatolGastroenterology hepatology 14, 141–142. 10.1038/nrgastro.2017.7 28174418

[B7] Eisenhauer E. A. Therasse P. Bogaerts J. Schwartz L. H. Sargent D. Ford R. (2009). New Response Evaluation Criteria in Solid Tumours: Revised RECIST Guideline (Version 1.1). Eur. J. Cancer 45, 228–247. 10.1016/j.ejca.2008.10.026 19097774

[B8] El-Khoueiry A. B. Sangro B. Yau T. Crocenzi T. S. Kudo M. Hsu C. (2017). Nivolumab in Patients with Advanced Hepatocellular Carcinoma (CheckMate 040): An Open-Label, Non-comparative, Phase 1/2 Dose Escalation and Expansion Trial. Lancet 389, 2492–2502. 10.1016/s0140-6736(17)31046-2 28434648 PMC7539326

[B9] Finn R. S. Merle P. Granito A. Huang Y. H. Bodoky G. Pracht M. (2018). Outcomes of Sequential Treatment with Sorafenib Followed by Regorafenib for HCC: Additional Analyses from the Phase III RESORCE Trial. J. Hepatol. 69, 353–358. 10.1016/j.jhep.2018.04.010 29704513

[B10] Heo Y. A. Syed Y. Y. (2018). Regorafenib: A Review in Hepatocellular Carcinoma. Drugs 78, 951–958. 10.1007/s40265-018-0932-4 29915898

[B11] Huang A. Yang X. R. Chung W. Y. Dennison A. R. Zhou J. (2020). Targeted Therapy for Hepatocellular Carcinoma. Signal Transduct. Target Ther. 5, 146. 10.1038/s41392-020-00264-x 32782275 PMC7419547

[B12] Kirstein M. M. Scheiner B. Marwede T. Wolf C. Voigtländer T. Semmler G. (2020). Sequential Systemic Treatment in Patients with Hepatocellular Carcinoma. Aliment. Pharmacol. Ther. 52, 205–212. 10.1111/apt.15789 32432799

[B13] Kudo M. (2018). Systemic Therapy for Hepatocellular Carcinoma: Latest Advances. Cancers 10 (11), 412. 10.3390/cancers10110412 PMC6266463 30380773

[B14] Liccioni A. Reig M. Bruix J. (2014). Treatment of Hepatocellular Carcinoma. Dig. Dis. 32, 554–563. 10.1159/000360501 25034288

[B15] Liu P. H. Huo T. I. Miksad R. A. (2018). Hepatocellular Carcinoma with Portal Vein Tumor Involvement: Best Management Strategies. Semin. Liver Dis. 38, 242–251. 10.1055/s-0038-1666805 30041276

[B16] Ogasawara S. Ooka Y. Itokawa N. Inoue M. Okabe S. Seki A. (2020). Sequential Therapy with Sorafenib and Regorafenib for Advanced Hepatocellular Carcinoma: a Multicenter Retrospective Study in Japan. Invest New Drugs 38, 172–180. 10.1007/s10637-019-00801-8 31172442

[B17] Palmer D. H. (2008). Sorafenib in Advanced Hepatocellular Carcinoma. N. Engl. J. Med. 359, 2498–2499. author reply 2498-9. 19065750

[B18] Piscaglia F. Ogasawara S. (2018). Patient Selection for Transarterial Chemoembolization in Hepatocellular Carcinoma: Importance of Benefit/Risk Assessment. Liver cancer 7, 104–119. 10.1159/000485471 29662837 PMC5892363

[B19] Reig M. Rimola J. Torres F. Darnell A. Rodriguez-Lope C. Forner A. (2013). Postprogression Survival of Patients with Advanced Hepatocellular Carcinoma: Rationale for Second-Line Trial Design. Hepatology 58, 2023–2031. 10.1002/hep.26586 23787822

[B20] Rich N. E. Yopp A. C. Singal A. G. (2017). Medical Management of Hepatocellular Carcinoma. J. Oncol. Pract. 13, 356–364. 10.1200/jop.2017.022996 28605614

[B21] Rimassa L. Danesi R. Pressiani T. Merle P. (2019). Management of Adverse Events Associated with Tyrosine Kinase Inhibitors: Improving Outcomes for Patients with Hepatocellular Carcinoma. Cancer Treat. Rev. 77, 20–28. 10.1016/j.ctrv.2019.05.004 31195212

[B22] Rizzo A. Nannini M. Novelli M. Dalia Ricci A. Scioscio V. D. Pantaleo M. A. (2020). Dose Reduction and Discontinuation of Standard-Dose Regorafenib Associated with Adverse Drug Events in Cancer Patients: a Systematic Review and Meta-Analysis. Ther. Adv. Med. Oncol. 12, 1758835920936932. 10.1177/1758835920936932 32684988 PMC7343359

[B23] Strumberg D. Schultheis B. (2012). Regorafenib for Cancer. Expert Opin. Investig. Drugs 21, 879–889. 10.1517/13543784.2012.684752 22577890

[B24] Subramonian D. Phanhthilath N. Rinehardt H. Flynn S. Huo Y. Zhang J. (2020). Regorafenib Is Effective against Neuroblastoma *In Vitro* and *In Vivo* and Inhibits the RAS/MAPK, PI3K/Akt/mTOR and Fos/Jun Pathways. Br. J. Cancer 123, 568–579. 10.1038/s41416-020-0905-8 32457362 PMC7434894

[B25] Terashima T. Yamashita T. Sunagozaka H. Arai K. Kawaguchi K. Kitamura K. (2018). Analysis of the Liver Functional Reserve of Patients with Advanced Hepatocellular Carcinoma Undergoing Sorafenib Treatment: Prospects for Regorafenib Therapy. Hepatol. Res. 48, 956–966. 10.1111/hepr.13196 29845710

[B26] Truty M. J. Vauthey J. N. (2010). Surgical Resection of High-Risk Hepatocellular Carcinoma: Patient Selection, Preoperative Considerations, and Operative Technique. Ann. Surg. Oncol. 17, 1219–1225. 10.1245/s10434-010-0976-5 20405326 PMC4103783

[B27] Tsai A. K. Khan A. Y. Worgo C. E. Wang L. L. Liang Y. Davila E. (2017). A Multikinase and DNA-PK Inhibitor Combination Immunomodulates Melanomas, Suppresses Tumor Progression, and Enhances Immunotherapies. Cancer Immunol. Res. 5, 790–803. 10.1158/2326-6066.Cir-17-0009 28775208 PMC5626455

[B28] Uchikawa S. Kawaoka T. Aikata H. Kodama K. Nishida Y. Inagaki Y. (2018). Clinical Outcomes of Sorafenib Treatment Failure for Advanced Hepatocellular Carcinoma and Candidates for Regorafenib Treatment in Real-World Practice. Hepatol. Res. 48, 814–820. 10.1111/hepr.13180 29682855

[B29] Von Felden J. (2020). New Systemic Agents for Hepatocellular Carcinoma: an Update 2020. Curr. Opin. Gastroenterol. 36, 177–183. 10.1097/mog.0000000000000626 32101985

[B30] Wilhelm S. M. Dumas J. Adnane L. Lynch M. Carter C. A. Schütz G. (2011). Regorafenib (BAY 73-4506): a New Oral Multikinase Inhibitor of Angiogenic, Stromal and Oncogenic Receptor Tyrosine Kinases with Potent Preclinical Antitumor Activity. Int. J. Cancer 129, 245–255. 10.1002/ijc.25864 21170960

[B31] Wu R. Y. Kong P. F. Xia L. P. Huang Y. Li Z. L. Tang Y. Y. (2019). Regorafenib Promotes Antitumor Immunity via Inhibiting PD-L1 and Ido1 Expression in Melanoma. Clin. Cancer Res. 25, 4530–4541. 10.1158/1078-0432.Ccr-18-2840 30940655

[B32] Yeo W. Mok T. S. Zee B. Leung T. W. Lai P. B. Lau W. Y. (2005). A Randomized Phase III Study of Doxorubicin versus Cisplatin/interferon Alpha-2b/doxorubicin/fluorouracil (PIAF) Combination Chemotherapy for Unresectable Hepatocellular Carcinoma. J. Natl. Cancer Inst. 97, 1532–1538. 10.1093/jnci/dji315 16234567

